# Supplementary material for: Life in a Central European warm-temperate to subtropical open forest: Paleoecology of the rhinocerotids from Ulm-Westtangente (Aquitanian, Early Miocene, Germany)
Source: Naturwissenschaften. 2024 Feb 14;111(1):10. doi: 10.1007/s00114-024-01893-w (PMC11401789; doi:10.1007/s00114-024-01893-w)
Supplement: Supplementary file 3 — Supplementary file3 (PDF 213 MB) [file 114_2024_1893_MOESM3_ESM.pdf]

Ulm-Westangente (SNMS, Stuttgart)

PALEVOPRIM - October 2022 - Manon HULLOT
PALEVOPRIM - October 2023 - Jérôme SURAULT (acquisition), Manon HULLOT (treatment)

Mesaceratherium paulhiacense

SMNS-47926
SMNS-47947
SMNS-47954
SMNS-47978
SMNS-47990
SMNS-48071
SMNS-48098
SMNS-48174
SMNS-48126
SMNS-48108
SMNS-48139

Protaceratherium minutum

SMNS-47920
SMNS-47963
SMNS-47986
SMNS-47993
SMNS-48055
SMNS-48068
SMNS-48081
SMNS-48089
SMNS-48095
SMNS-48074

October 2023

SMNS-47871
SMNS-47876
SMNS-47977
SMNS-47980
SMNS-48104
SMNS-48107
SMNS-48128
SMNS-48132
SMNS-48134
SMNS-48164
SMNS-48168
SMNS-48176

PS8-Zinv-SNMS_47926-um3s-ptc-hsb

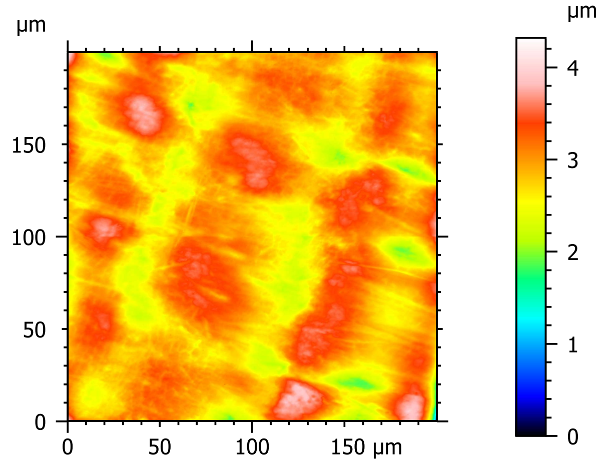

PS8-Zinv-SNMS_47926-um3s-ptc-hsb

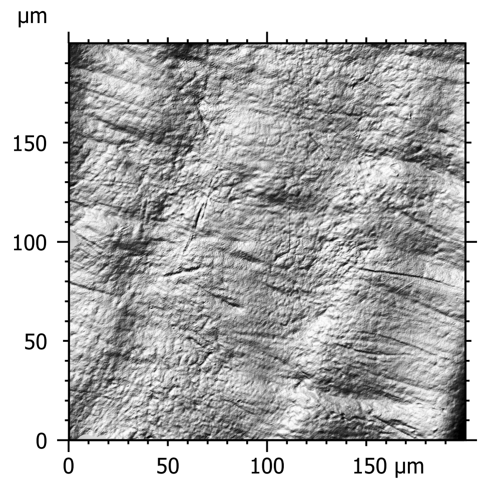

PS8-Zinv-SNMS_47926-um3s-ptc-nohsb

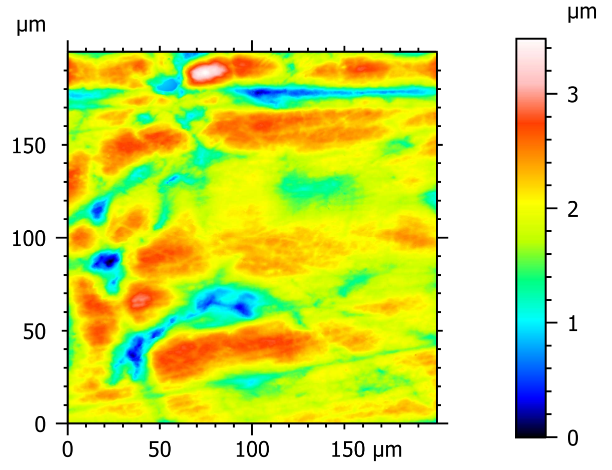

PS8-Zinv-SNMS_47926-um3s-ptc-nohsb

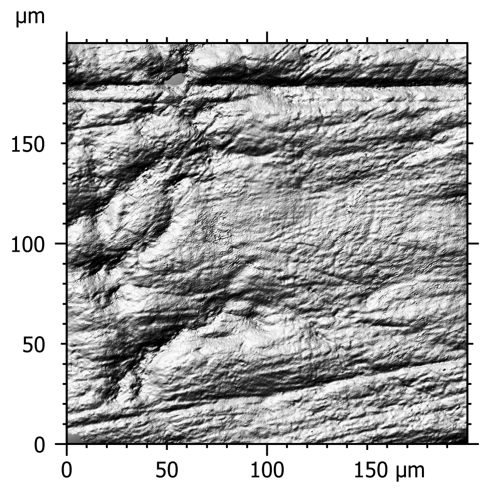

PS8-Zinv-SNMS_47947-um1s-ptc-hsb

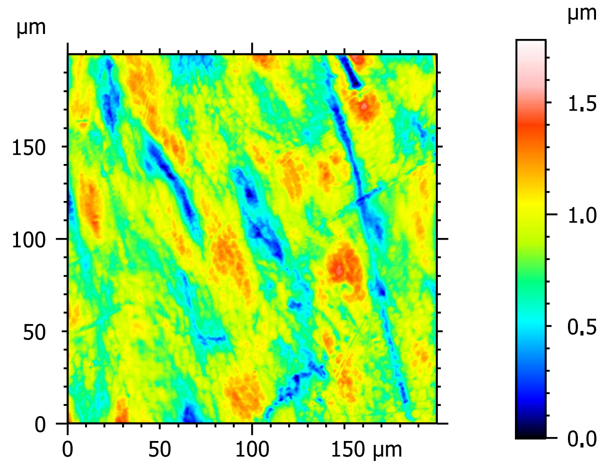

PS8-Zinv-SNMS_47947-um1s-ptc-hsb

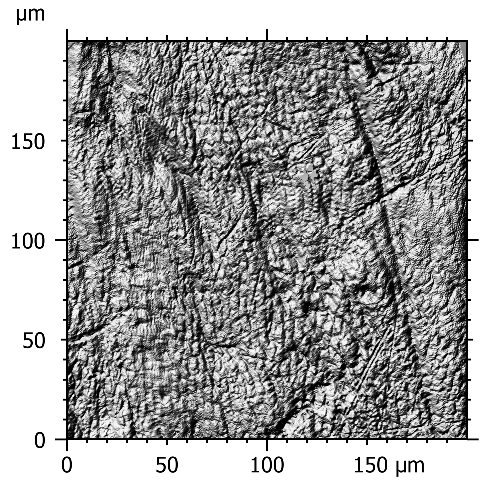

PS8-Zinv-SNMS_47954-lm2d-ptc-hsb

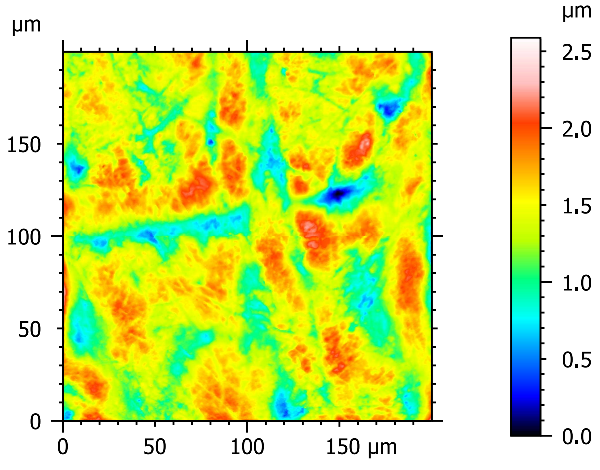

PS8-Zinv-SNMS_47954-lm2d-ptc-hsb

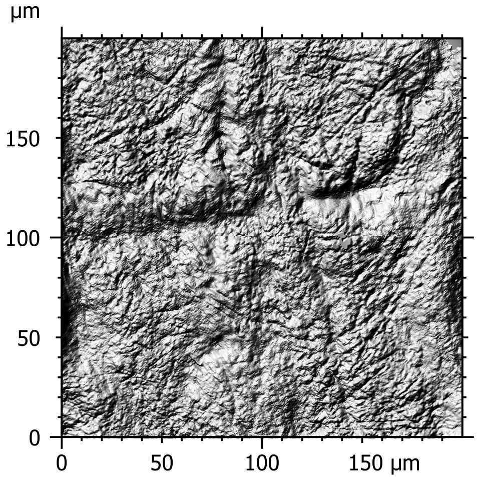

PS8-Zinv-SNMS_47978-lm2d-ptc-hsb

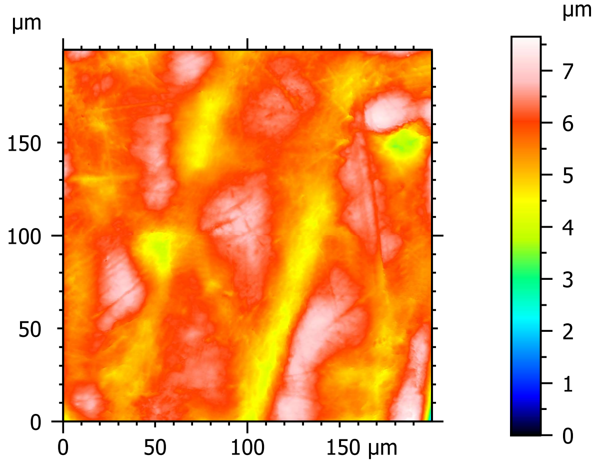

PS8-Zinv-SNMS_47978-lm2d-ptc-hsb

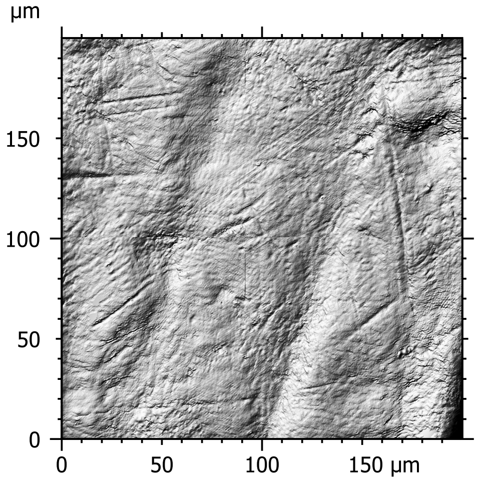

PS8-Zinv-SNMS_47978-lm2d-ptc-nohsb

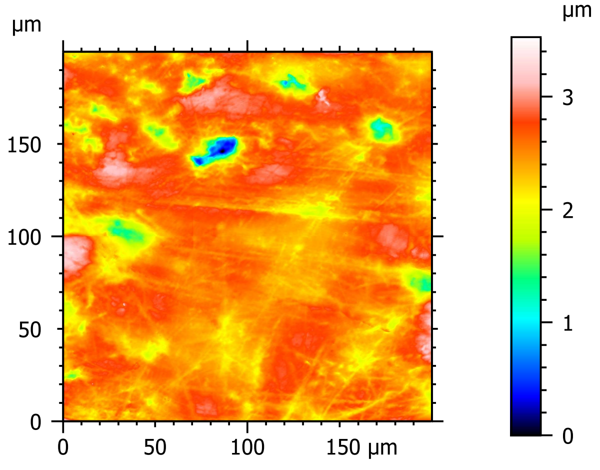

PS8-Zinv-SNMS_47978-lm2d-ptc-nohsb

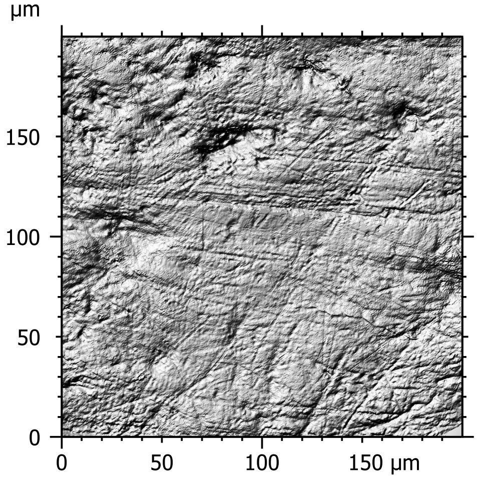

MountainsMap® Premium 10.0.10510

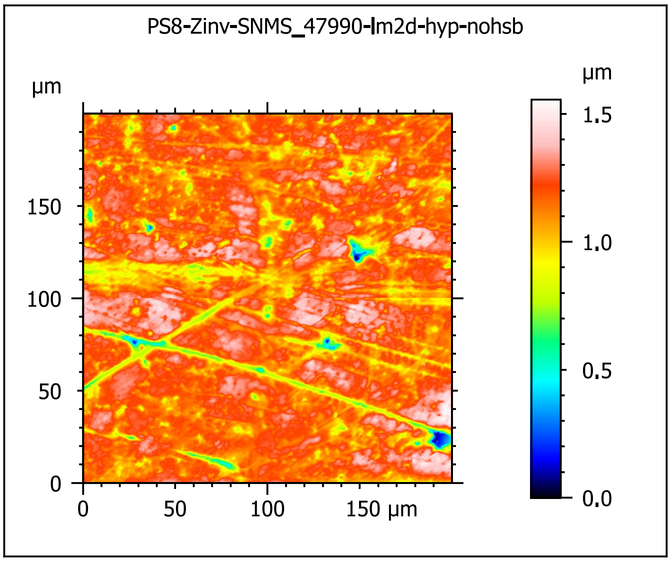

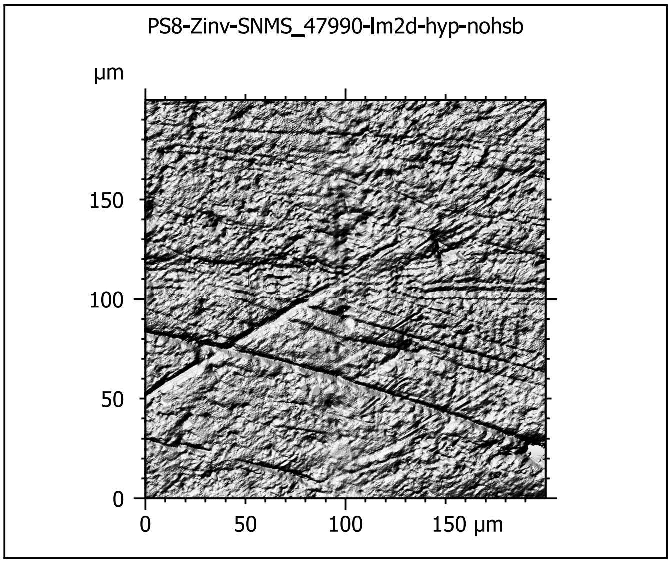

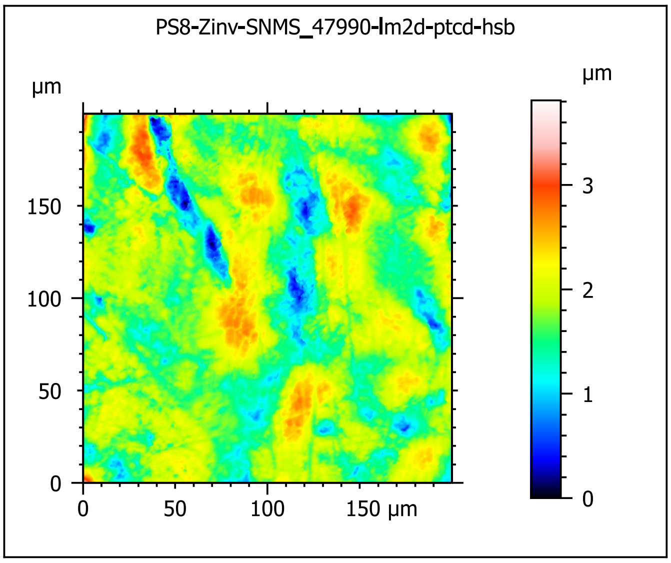

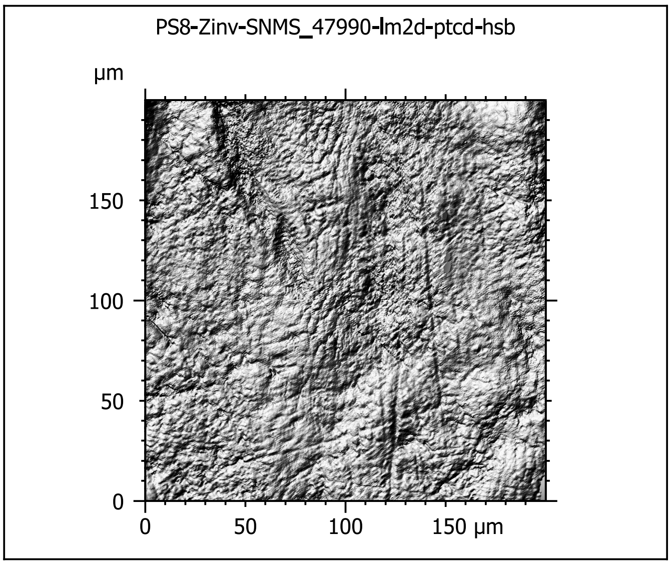

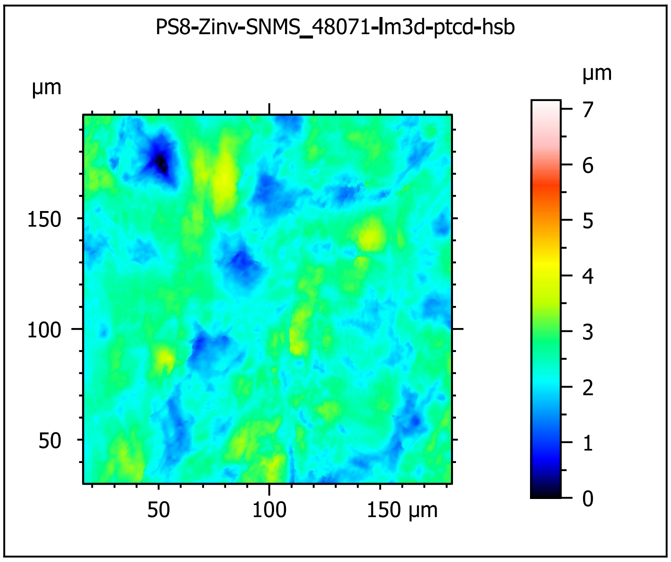

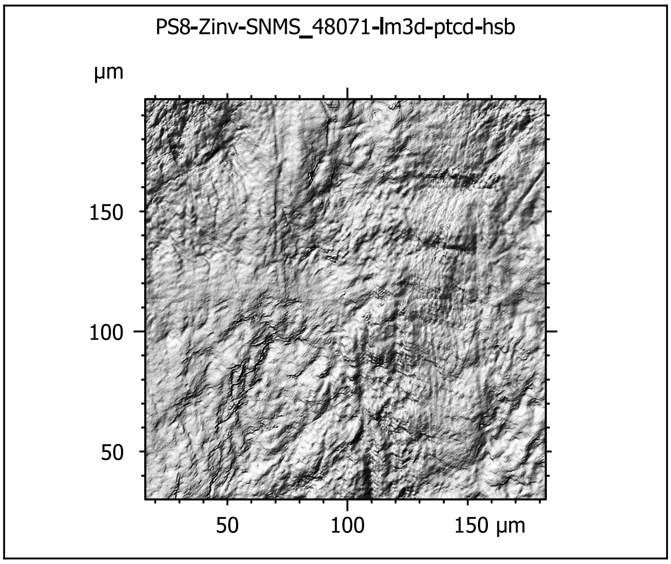

MountainsMap® Premium 10.0.10510

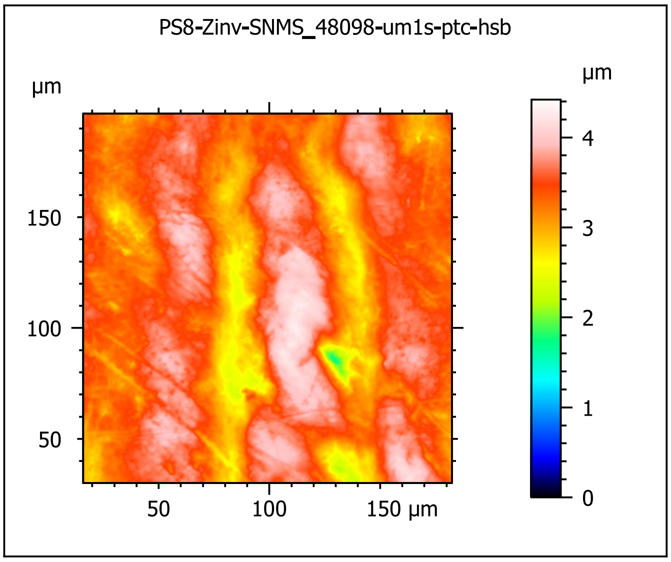

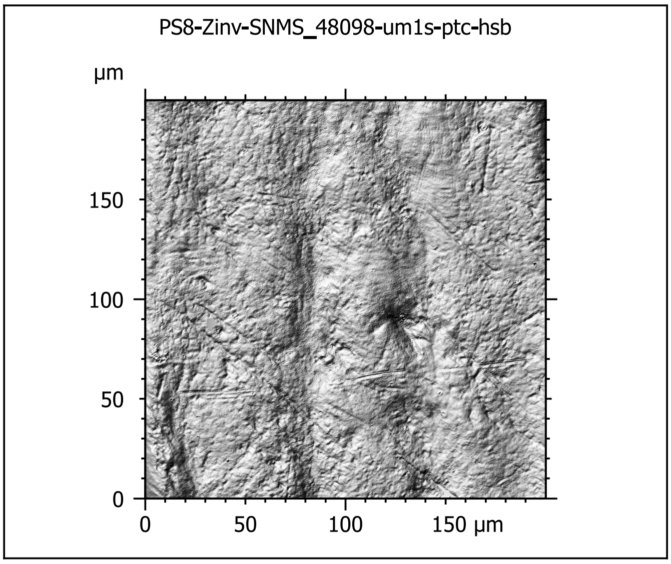

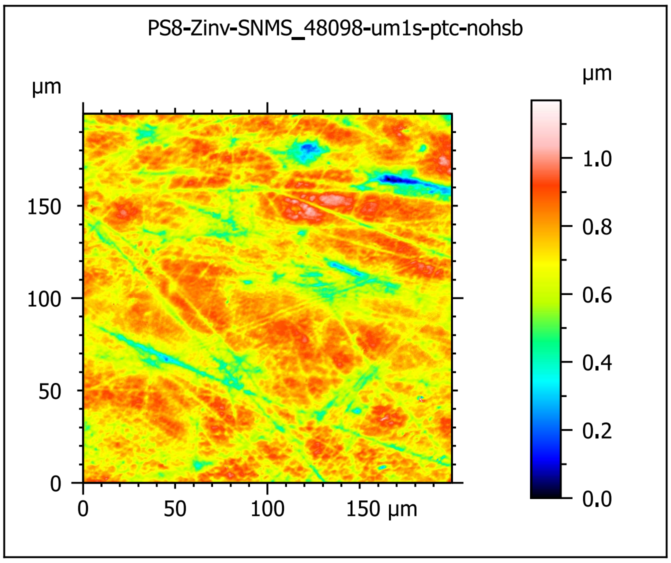

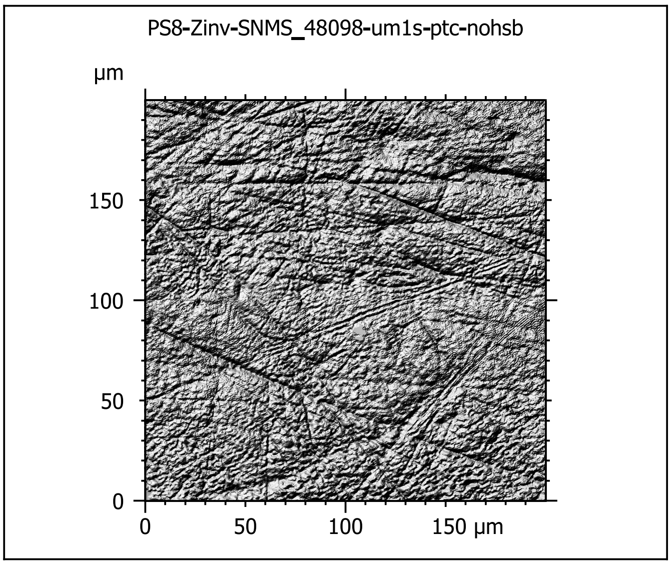

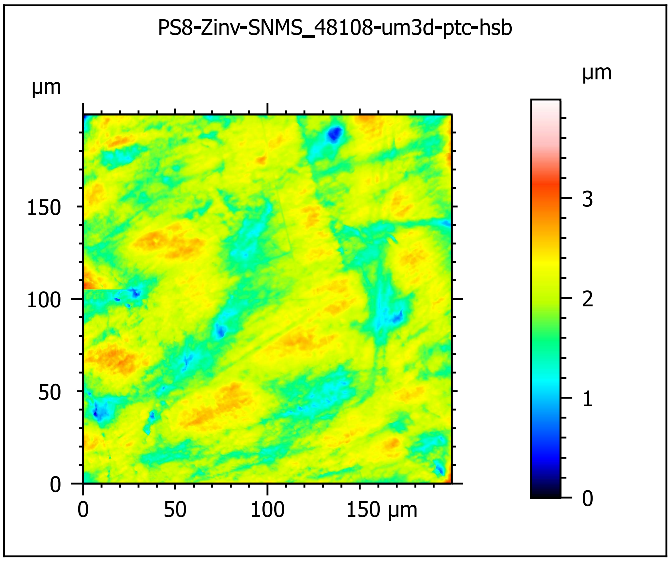

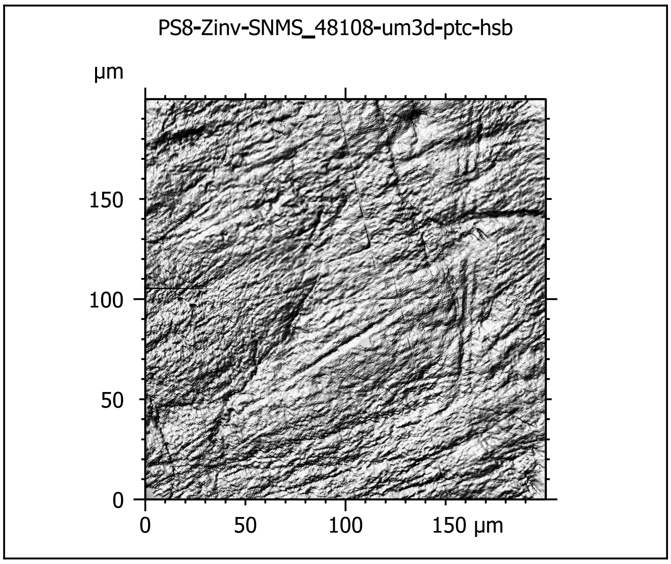

MountainsMap® Premium 10.0.10510

PS8-Zinv-SNMS_48126-um2s-ptc-hsb

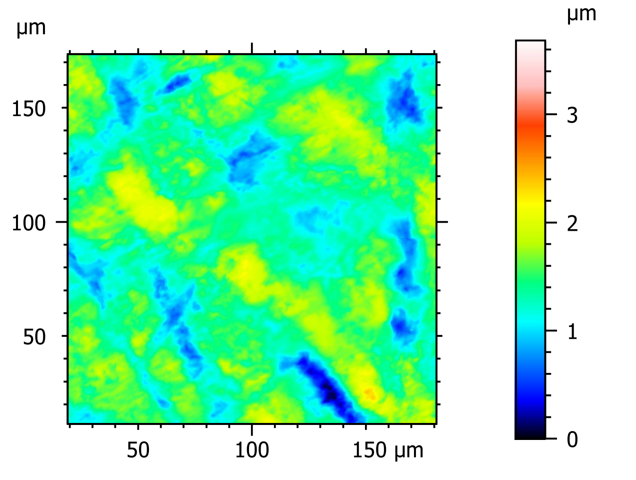

PS8-Zinv-SNMS_48126-um2s-ptc-hsb

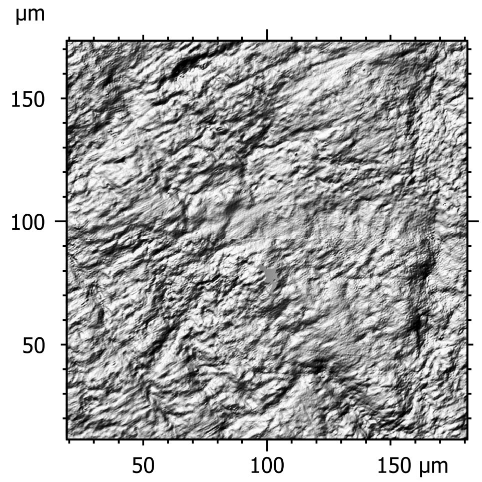

PS8-Zinv-SNMS_48139-um1d-ptc-hsb

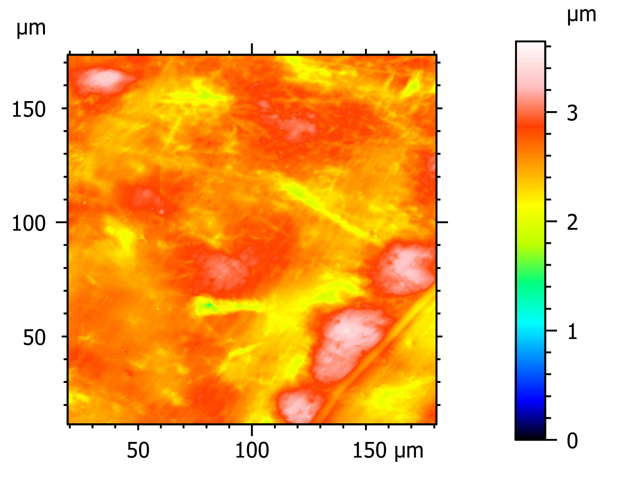

PS8-Zinv-SNMS_48139-um1d-ptc-hsb

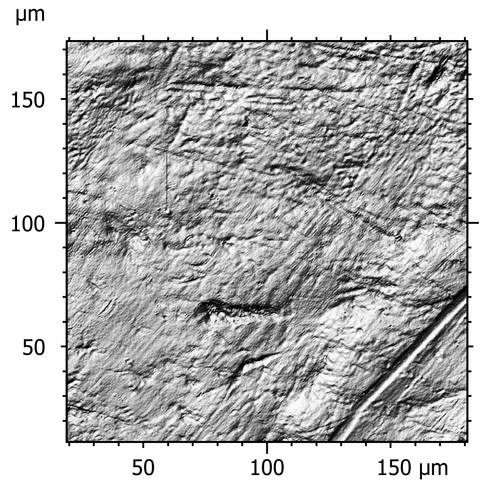

PS8-Zinv-SNMS_48174-um2d-ptc-nohsb

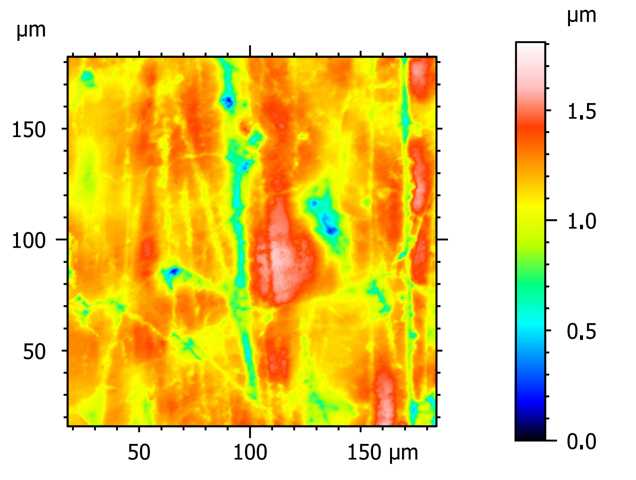

PS8-Zinv-SNMS_48174-um2d-ptc-nohsb

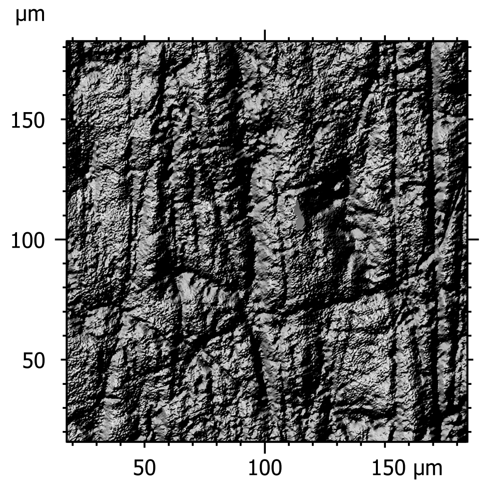

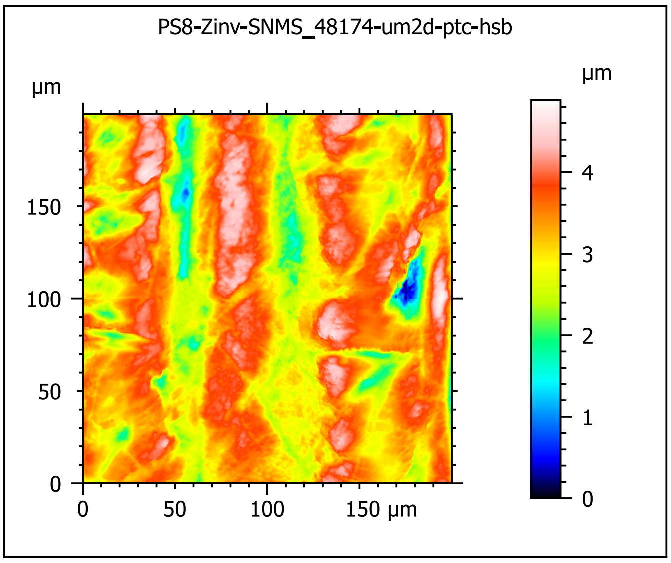
PS8-Zinv-SNMS_48174-um2d-ptc-hsb
µm
150
100
50
0
0
50
100
150 µm
µm
4
3
2
1
0

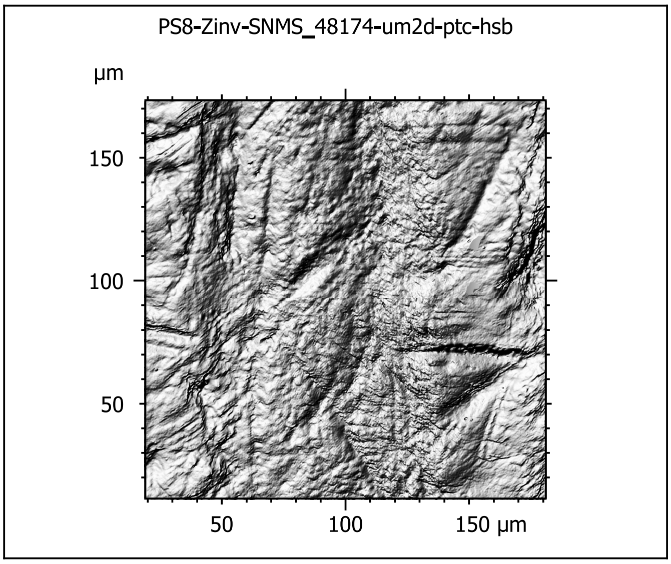
PS8-Zinv-SNMS_48174-um2d-ptc-hsb
µm
150
100
50
50
100
150 µm

PS8-Zinv-SNMS_47920-lm1s-ptcd-hsb

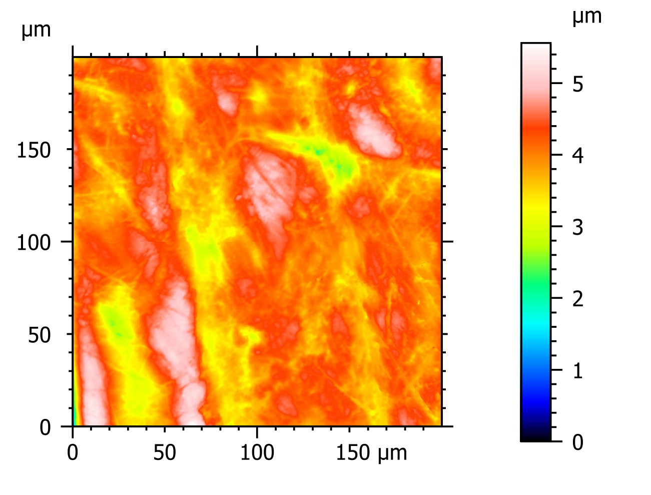

PS8-Zinv-SNMS_47920-lm1s-ptcd-hsb

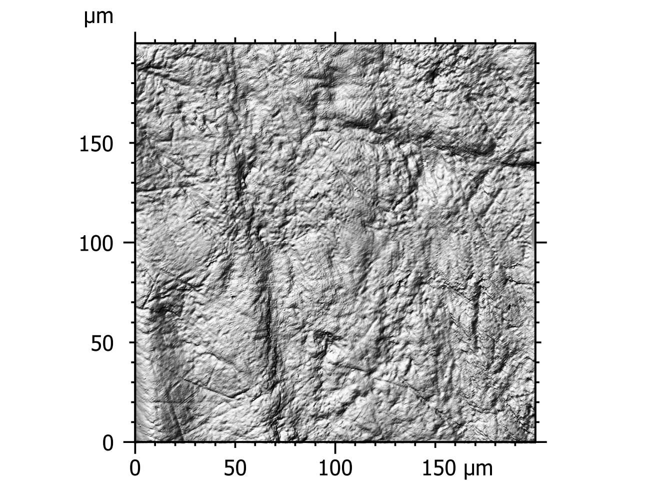

PS8-Zinv-SNMS_47963-um2s-ptc-hsb

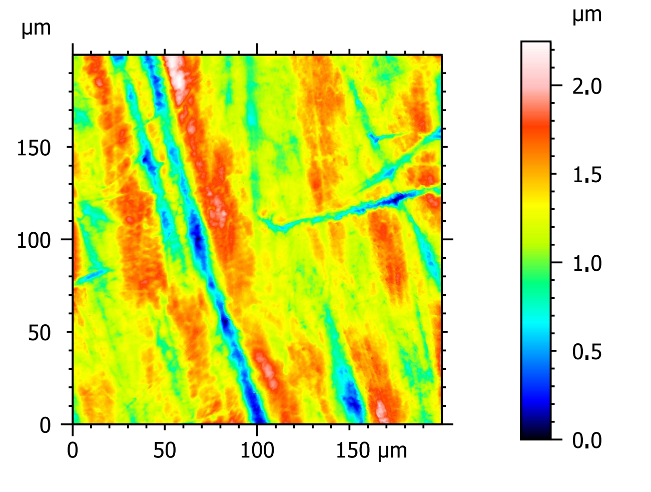

PS8-Zinv-SNMS_47963-um2s-ptc-hsb

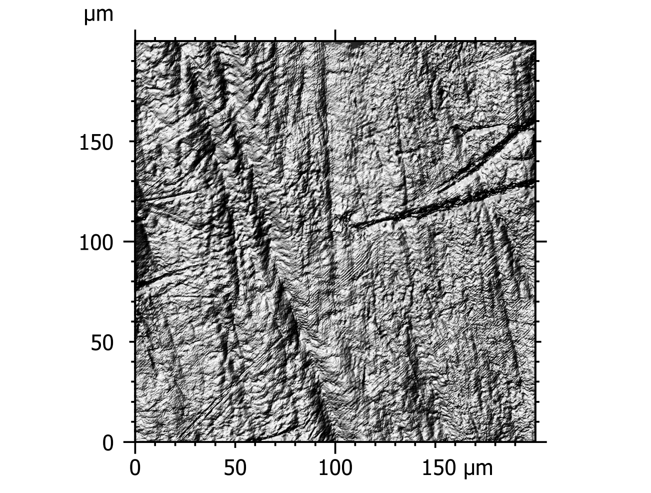

PS8-Zinv-SNMS_47986-lm1d-hyp-hsb

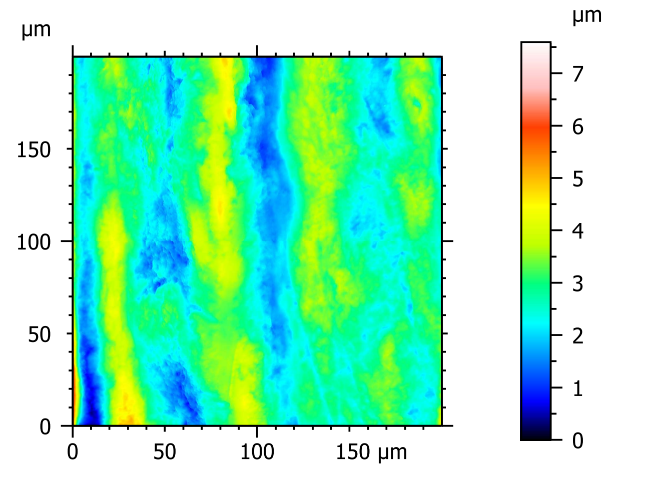

PS8-Zinv-SNMS_47986-lm1d-hyp-hsb

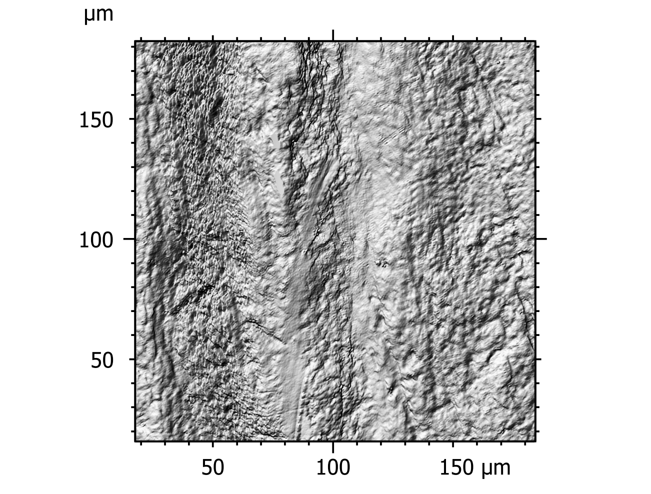

MountainsMap® Premium 10.0.10510

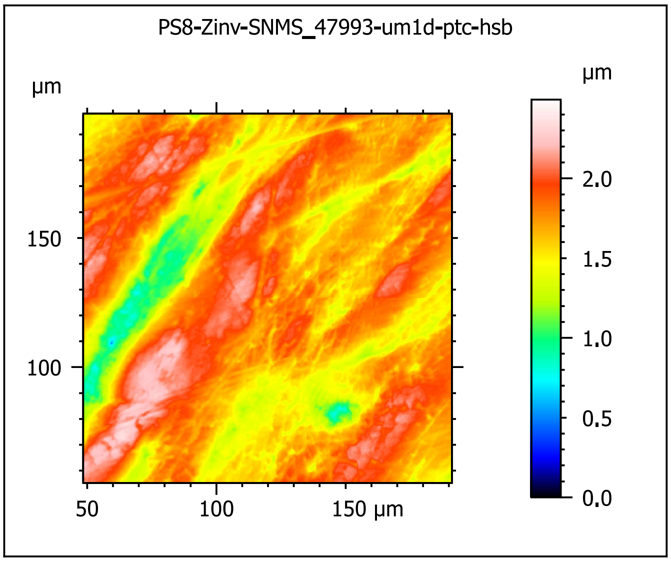

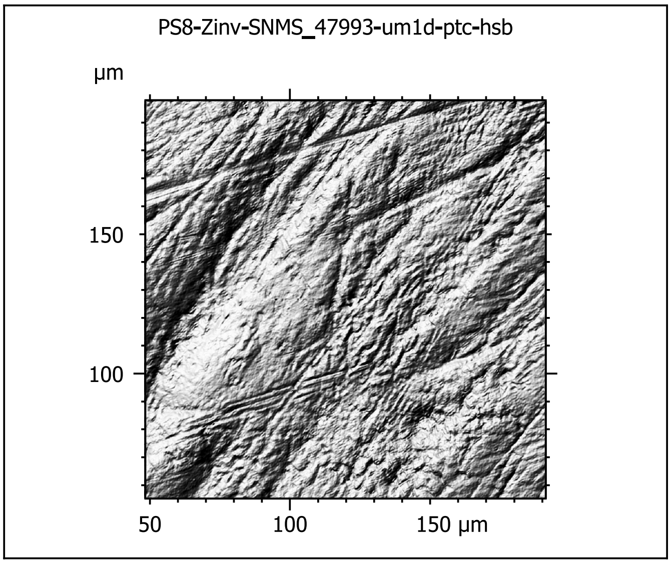

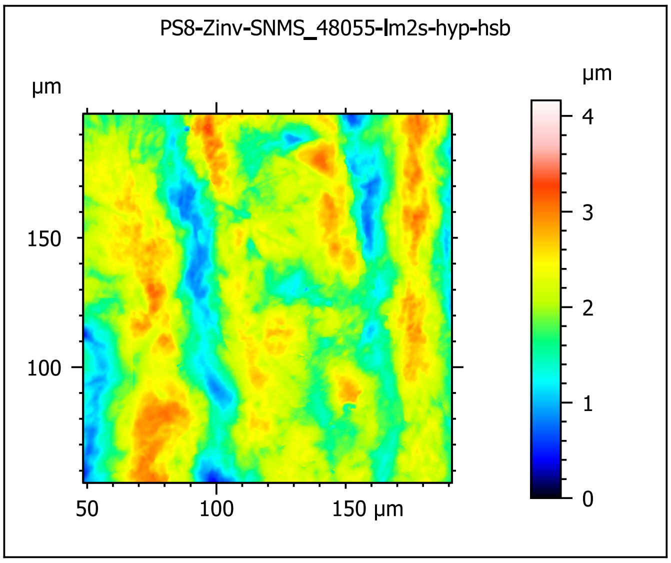

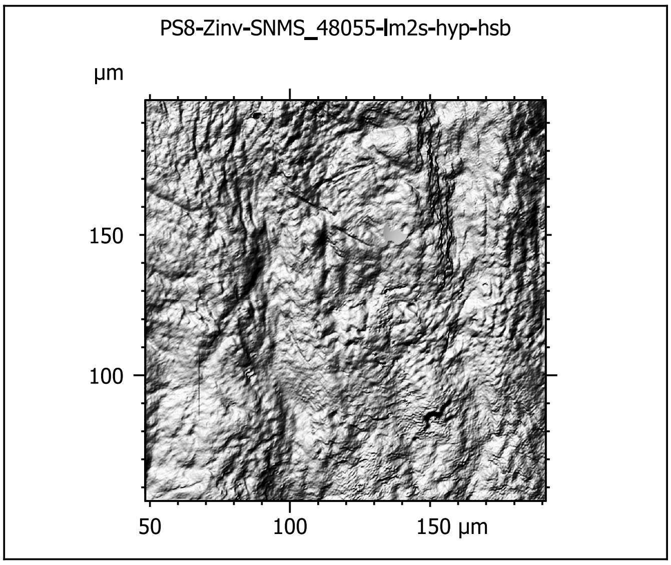

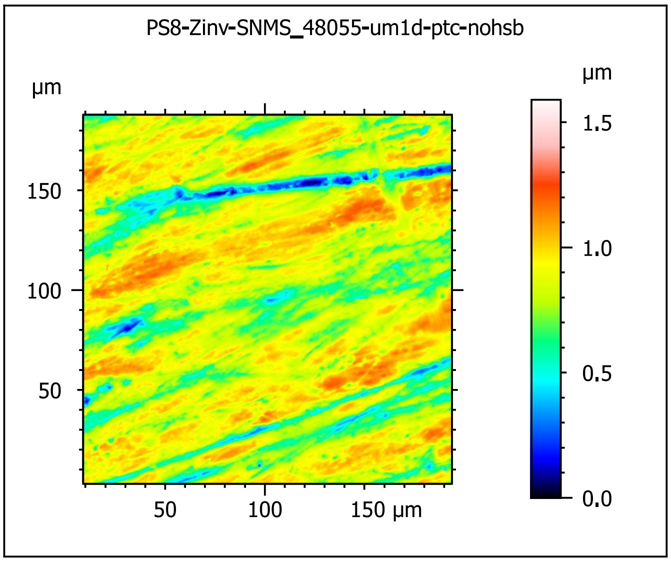

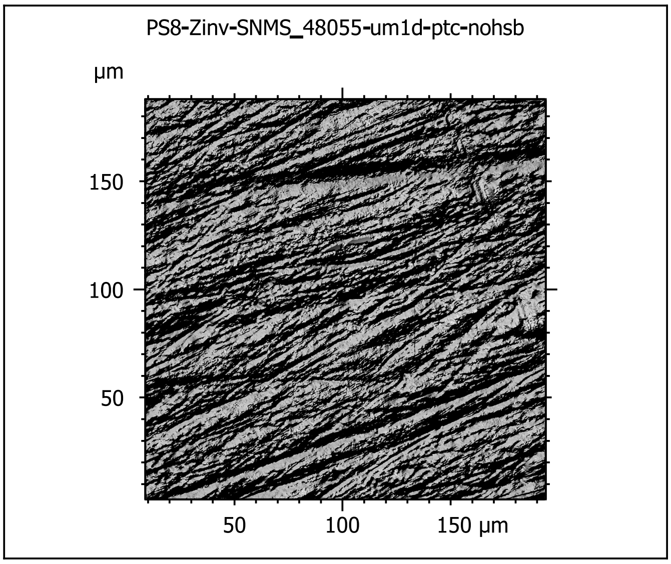

MountainsMap® Premium 10.0.10510

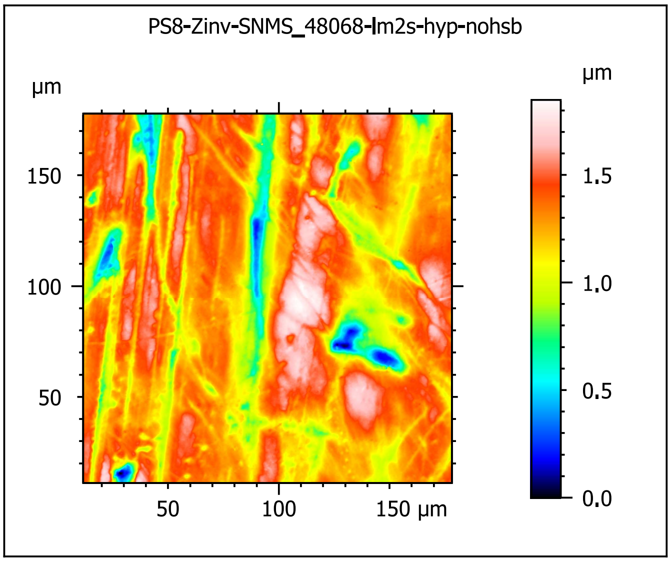

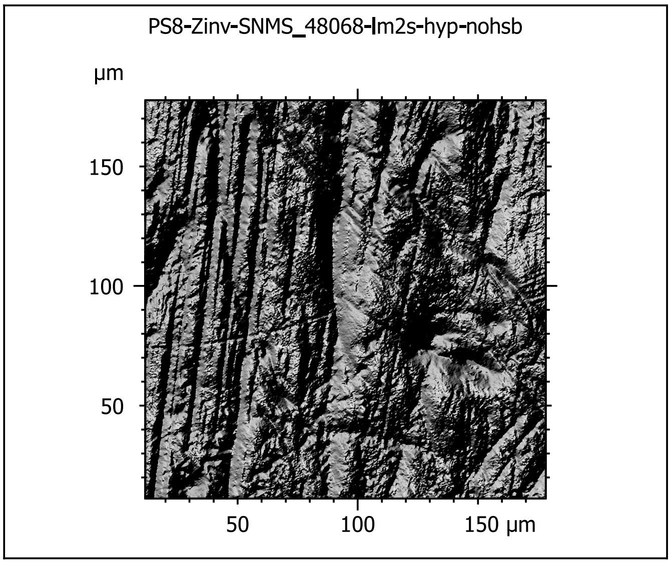

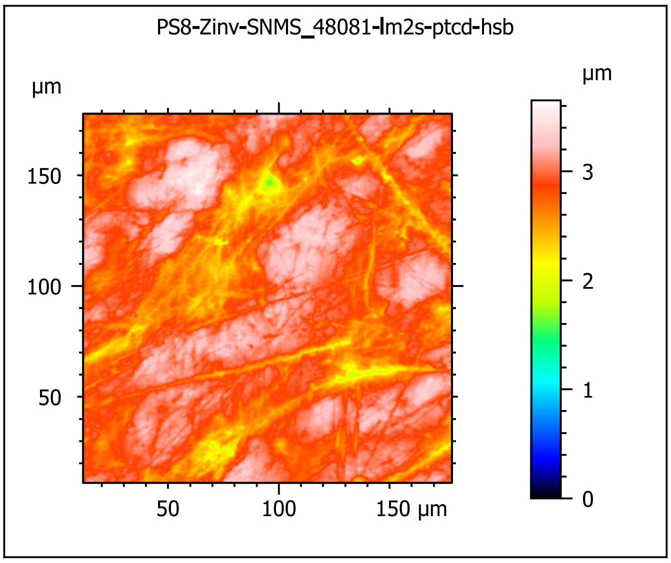

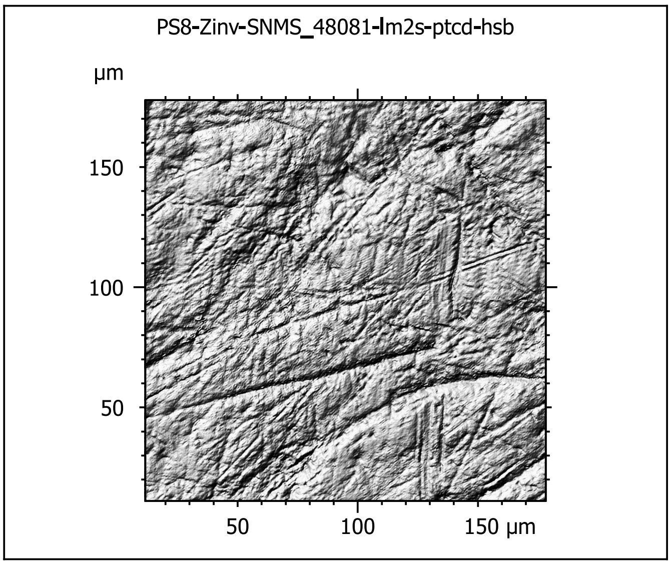

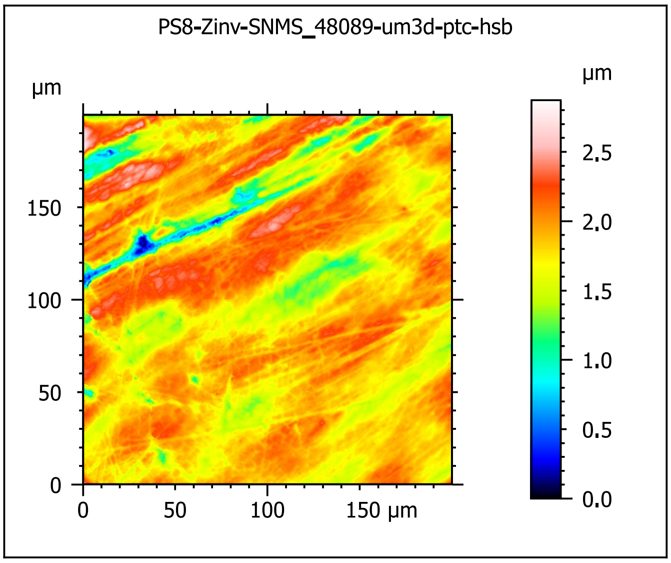

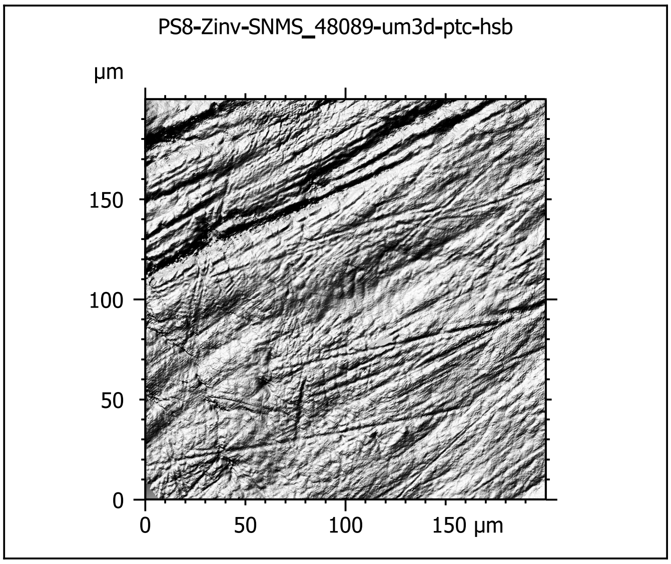

MountainsMap® Premium 10.0.10510

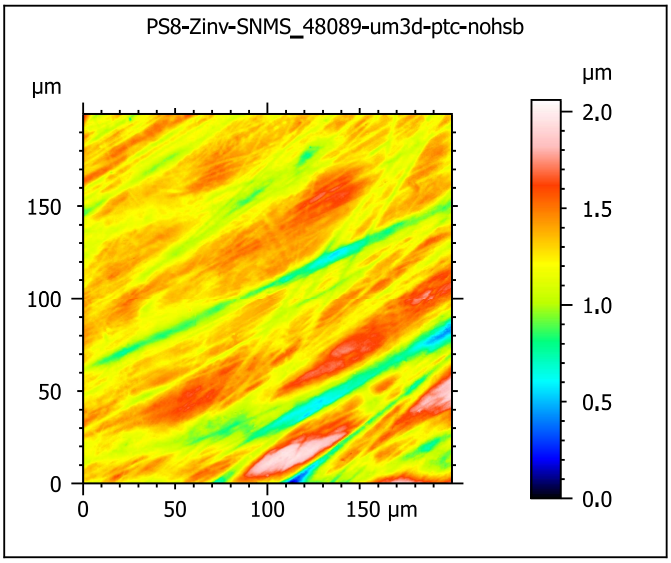
PS8-Zinv-SNMS_48089-um3d-ptc-nohsb
µm
0
50
100
150
0
50
100
150 µm
µm
2,0
1,5
1,0
0,5
0,0

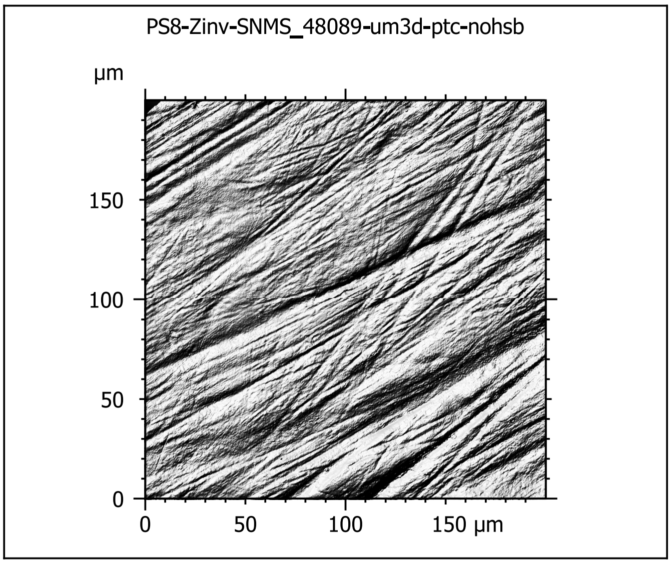
PS8-Zinv-SNMS_48089-um3d-ptc-nohsb
µm
0
50
100
150
0
50
100
150 µm

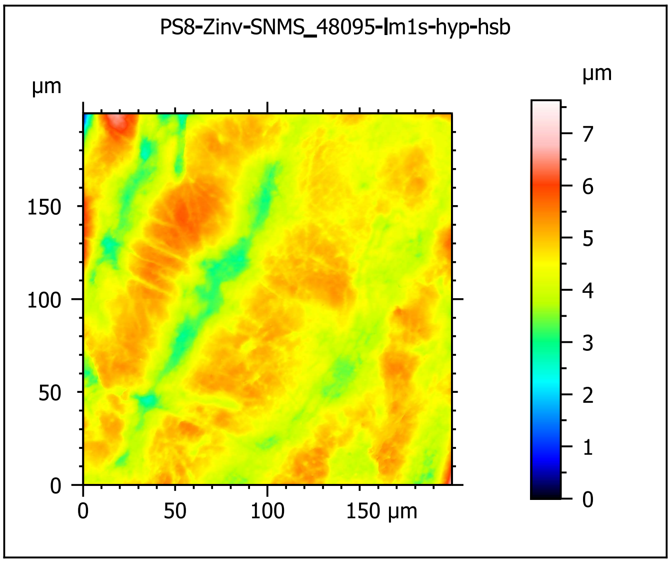
PS8-Zinv-SNMS_48095-lm1s-hyp-hsb
µm
0
50
100
150
0
50
100
150 µm
µm
7
6
5
4
3
2
1
0

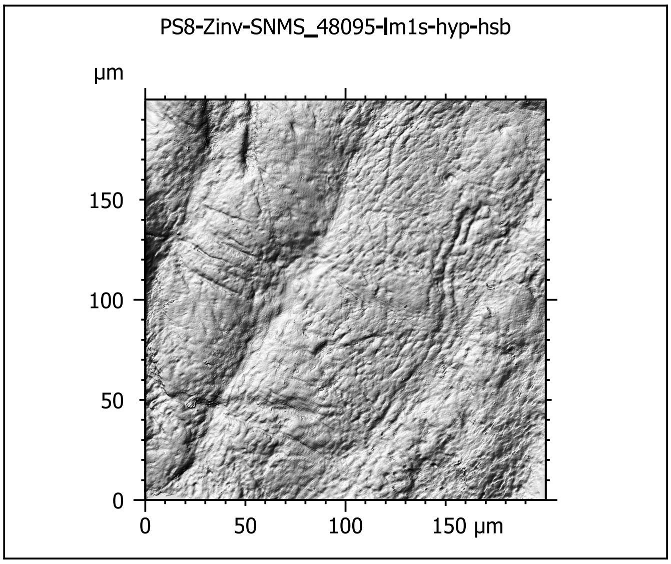
PS8-Zinv-SNMS_48095-lm1s-hyp-hsb
µm
0
50
100
150
0
50
100
150 µm

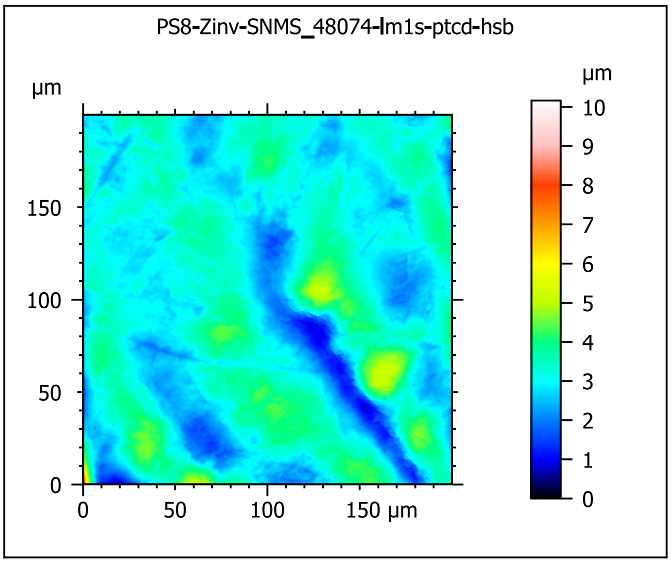
PS8-Zinv-SNMS_48074-lm1s-ptcd-hsb
µm
0
50
100
150
0
50
100
150 µm
µm
10
9
8
7
6
5
4
3
2
1
0

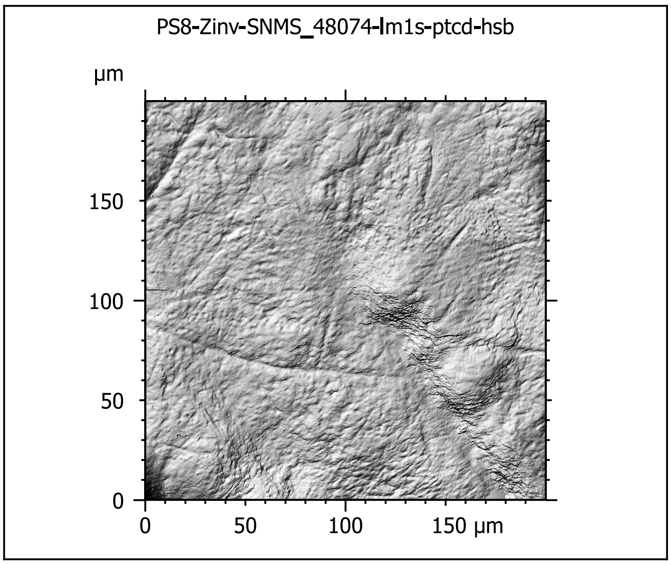
PS8-Zinv-SNMS_48074-lm1s-ptcd-hsb
µm
0
50
100
150
0
50
100
150 µm

PS8-Zinv-SNMS_47871-UM2d-ptc-hsb

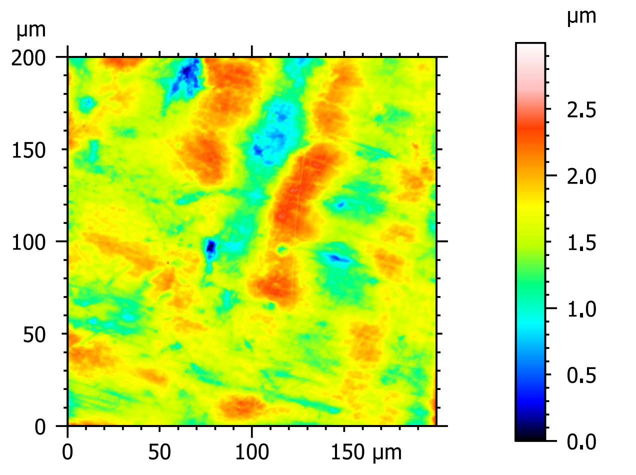

PS8-Zinv-SNMS_47871-UM2d-ptc-hsb

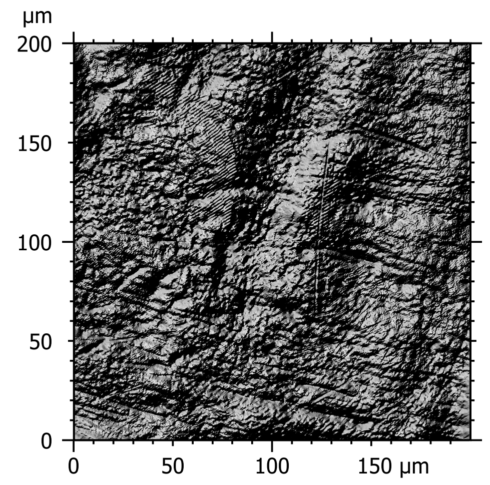

PS8-Zinv-SNMS_47871-UM2s-ptc-no-hsb

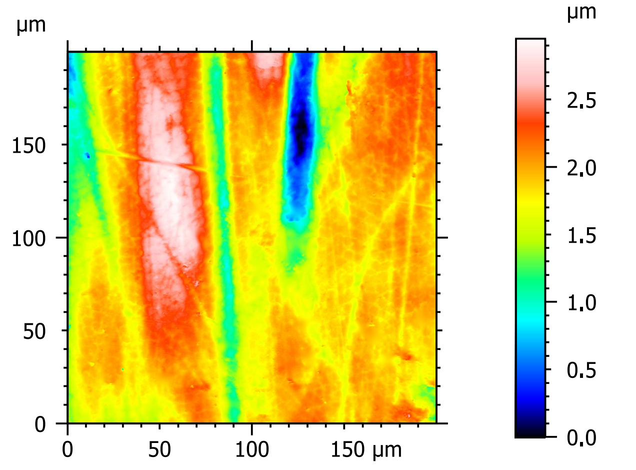

PS8-Zinv-SNMS_47871-UM2s-ptc-no-hsb

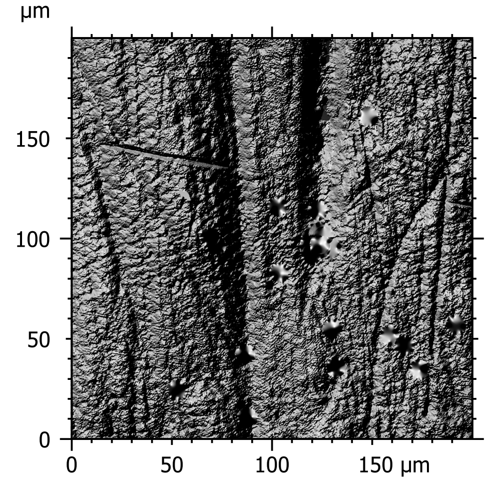

PS8-Zinv-SNMS_47876-UM2d-ptc-hsb

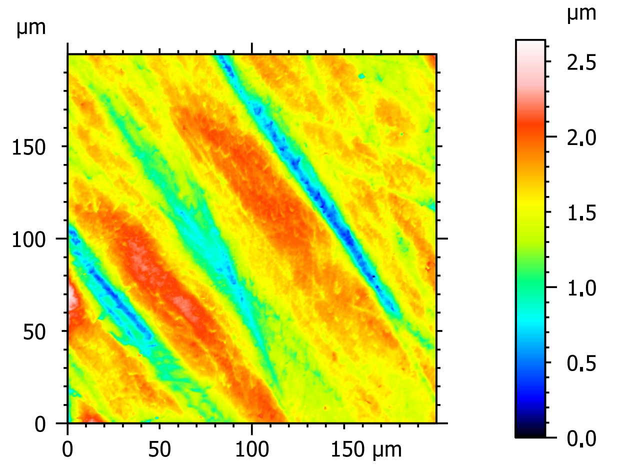

PS8-Zinv-SNMS_47876-UM2d-ptc-hsb

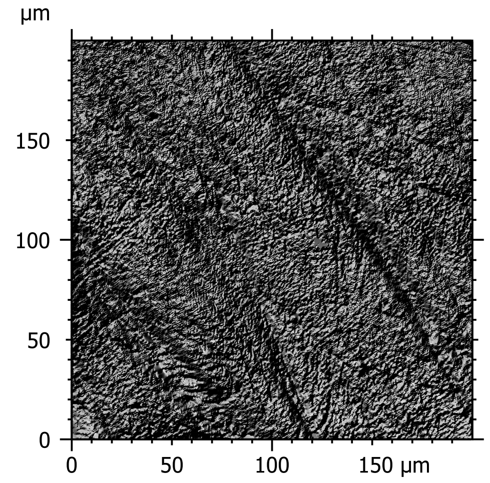

MountainsMap® Premium 10.0.10510

PS8-Zinv-SNMS_47876-UM2d-ptc-no-hsb

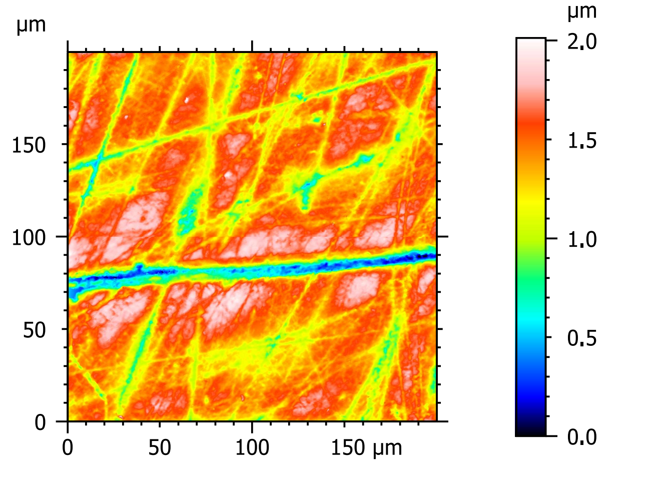

PS8-Zinv-SNMS_47876-UM2d-ptc-no-hsb

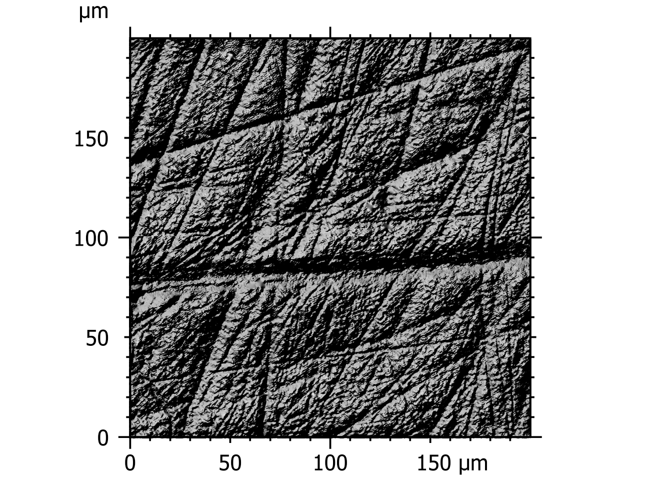

PS8-Zinv-SNMS_47977-UM1s-ptc-hsb

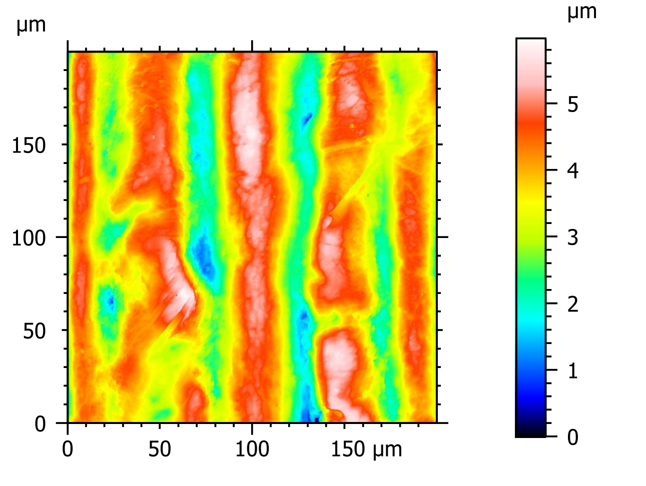

PS8-Zinv-SNMS_47977-UM1s-ptc-hsb

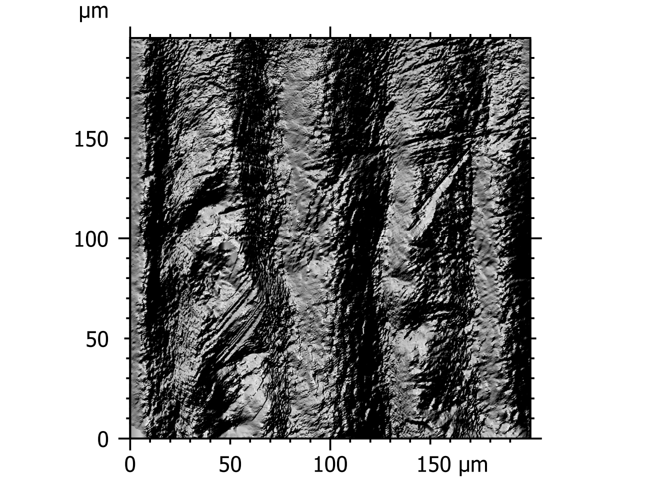

PS8-Zinv-SNMS_47980-lm2d-ptcd-hsb

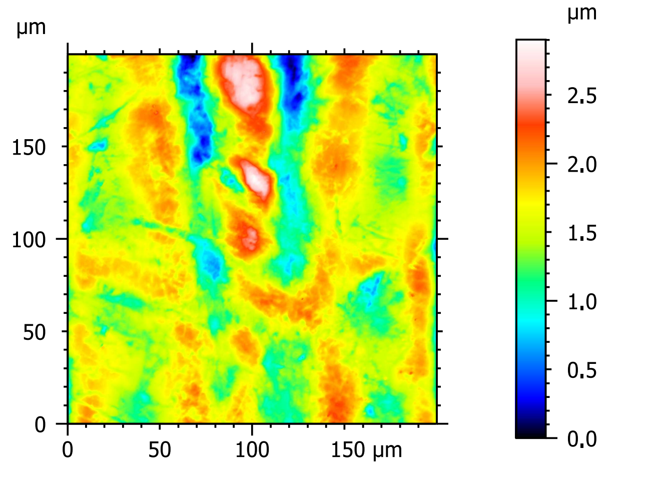

PS8-Zinv-SNMS_47980-lm2d-ptcd-hsb

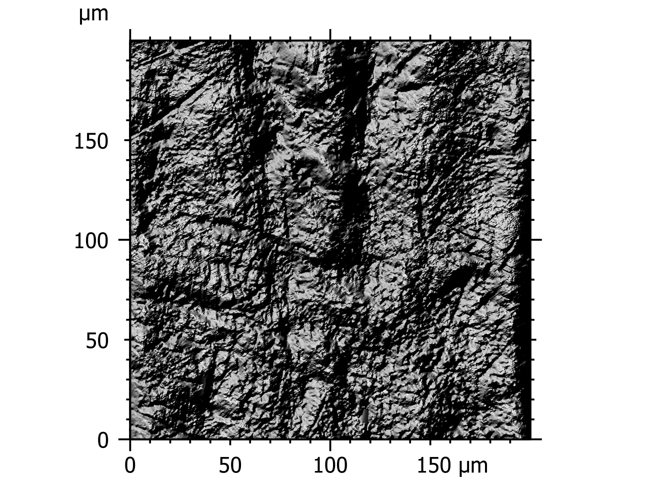

MountainsMap® Premium 10.0.10510

PS8-Zinv-SNMS_48104-lm2s-hypd-hsb

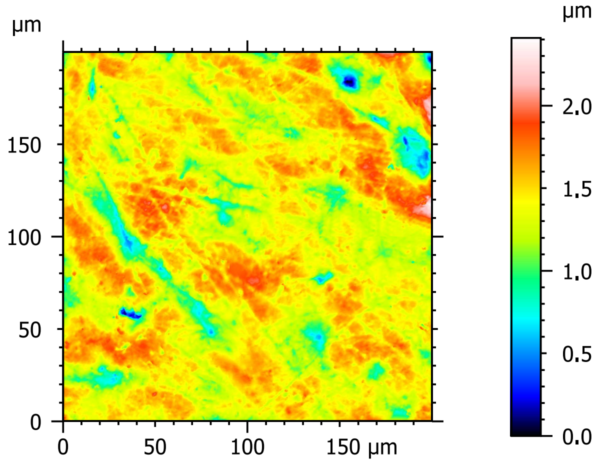

PS8-Zinv-SNMS_48104-lm2s-hypd-hsb

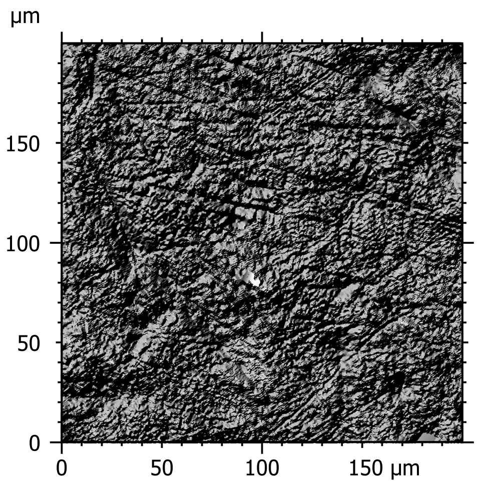

PS8-Zinv-SNMS_48107-lm2d-hypd-hsb

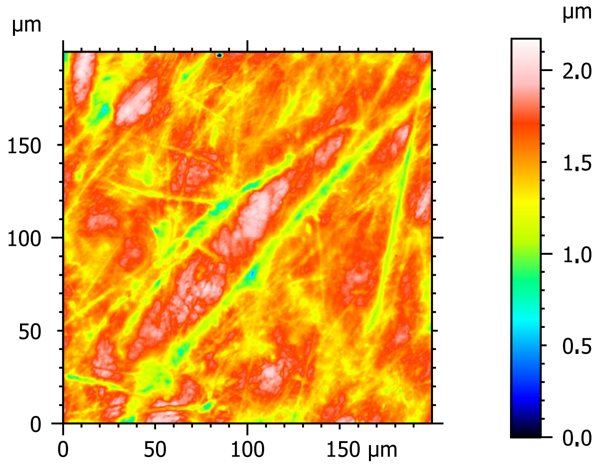

PS8-Zinv-SNMS_48107-lm2d-hypd-hsb

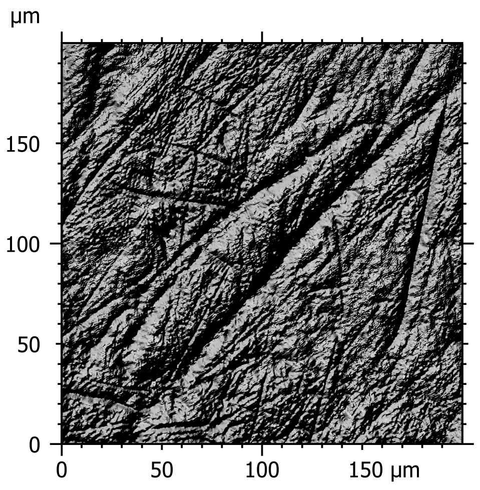

PS8-Zinv-SNMS_48107-lm2d-hypd-no-hsb

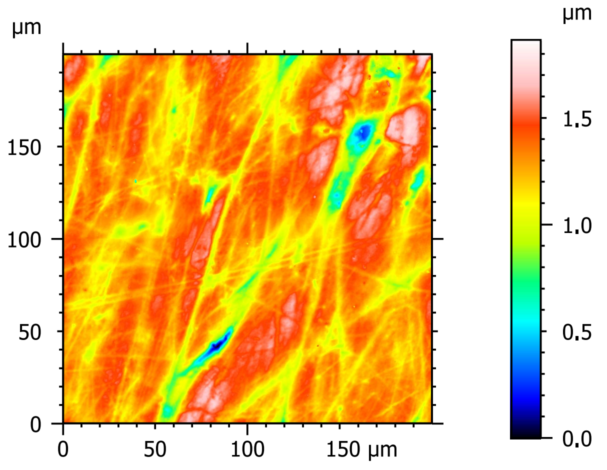

PS8-Zinv-SNMS_48107-lm2d-hypd-no-hsb

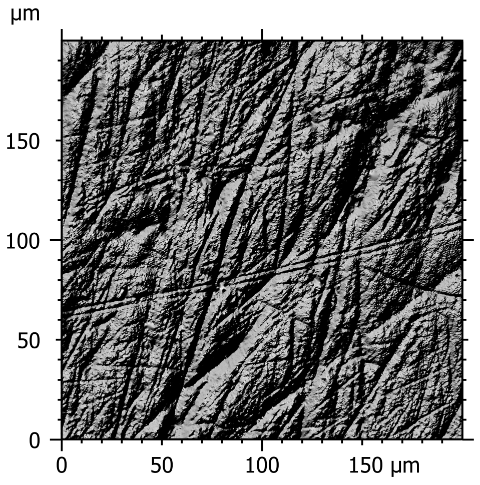

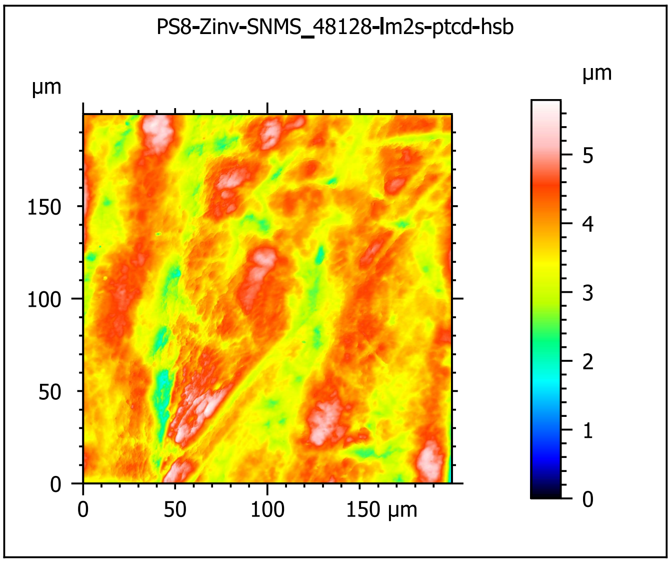

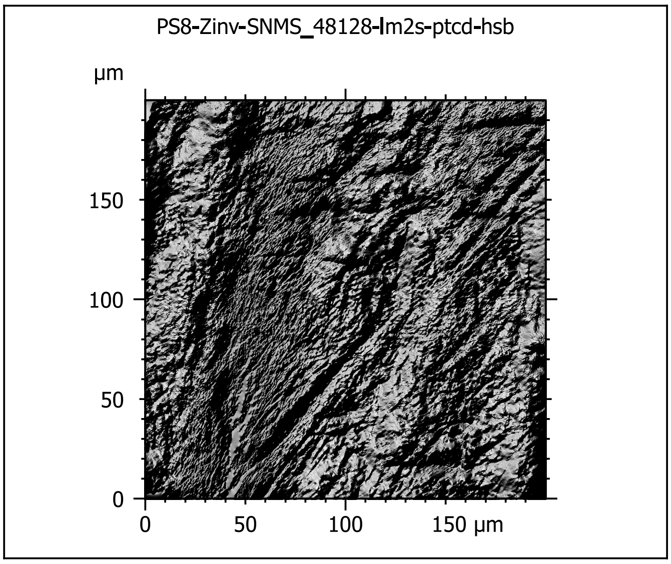

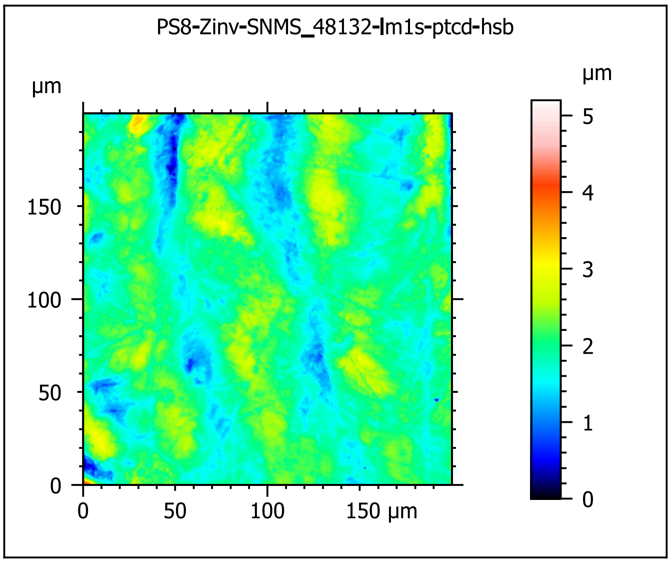

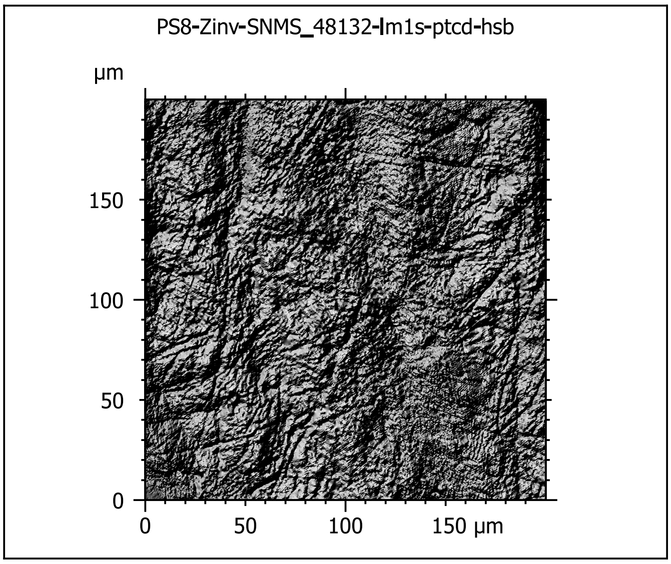

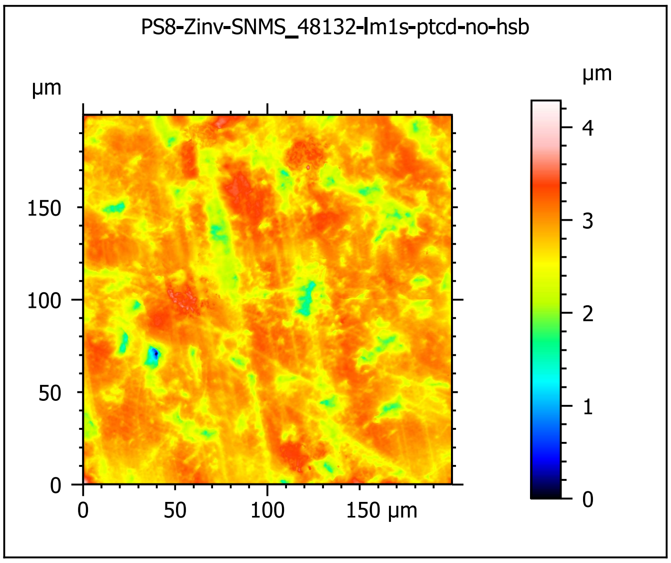

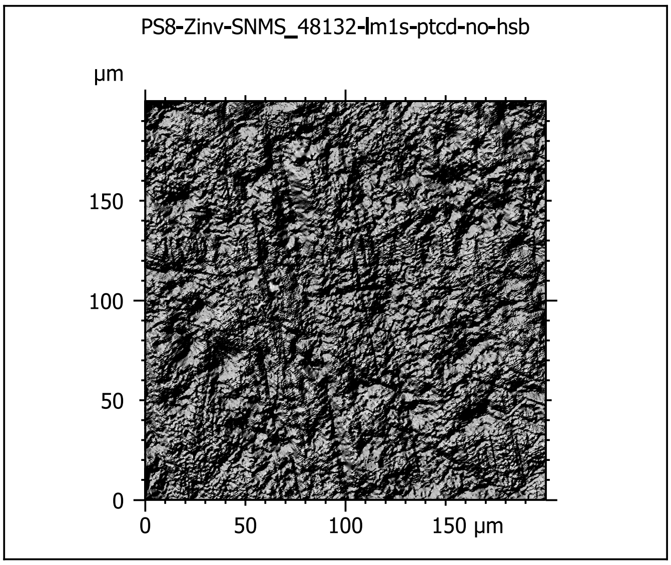

MountainsMap® Premium 10.0.10510

PS8-Zinv-SNMS_48134-lm1s-ptcd-hsb

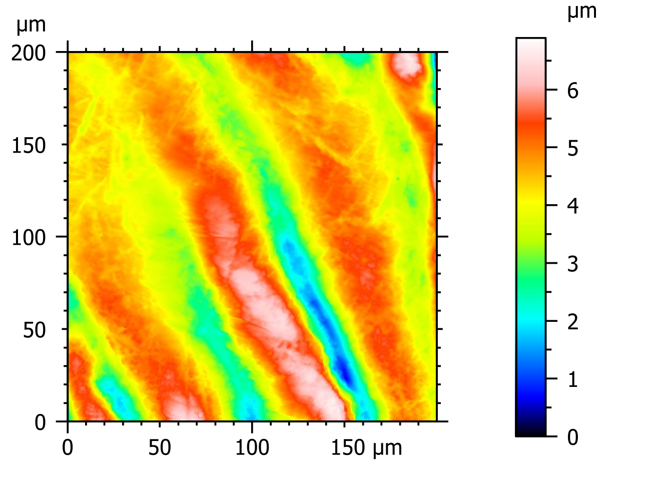

PS8-Zinv-SNMS_48134-lm1s-ptcd-hsb

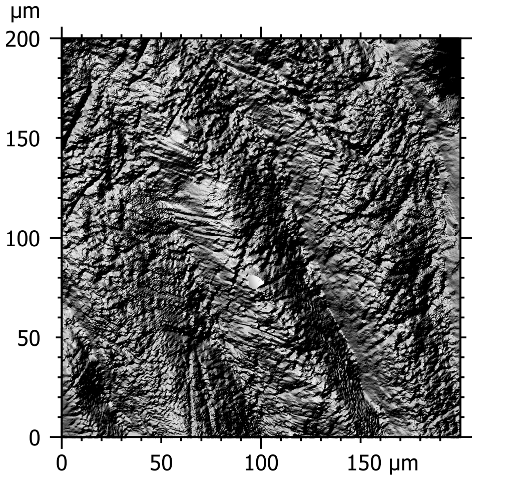

PS8-Zinv-SNMS_48163-UM2s-ptc-hsb

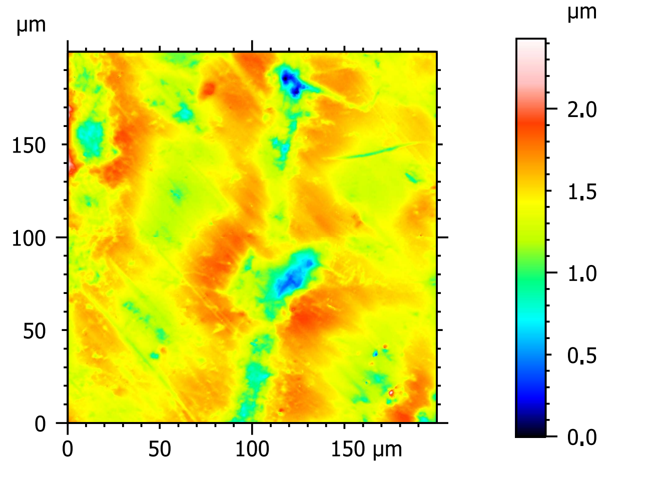

PS8-Zinv-SNMS_48163-UM2s-ptc-hsb

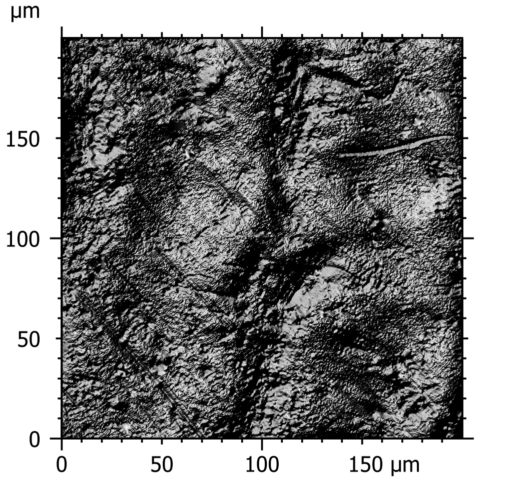

PS8-Zinv-SNMS_48164-UM2d-ptc-no-hsb

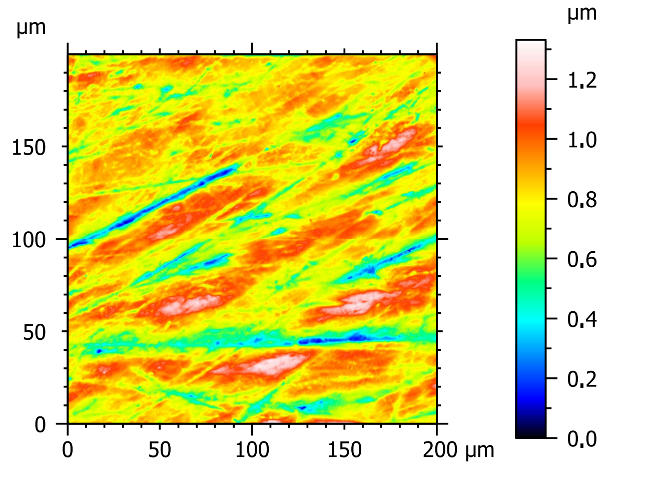

PS8-Zinv-SNMS_48164-UM2d-ptc-no-hsb

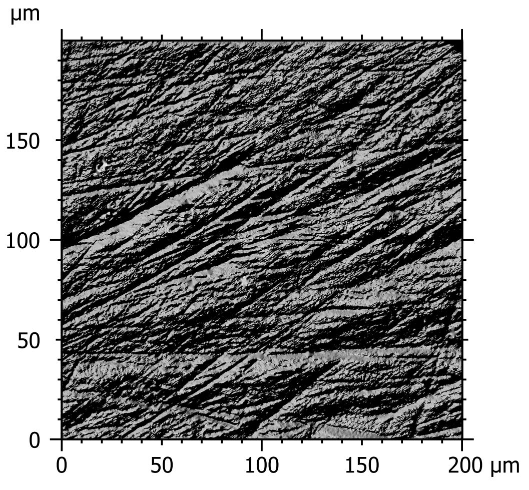

MountainsMap® Premium 10.0.10510

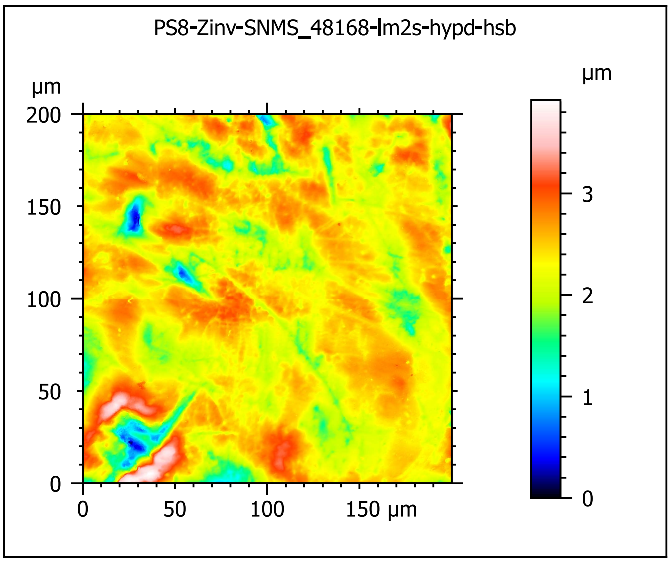

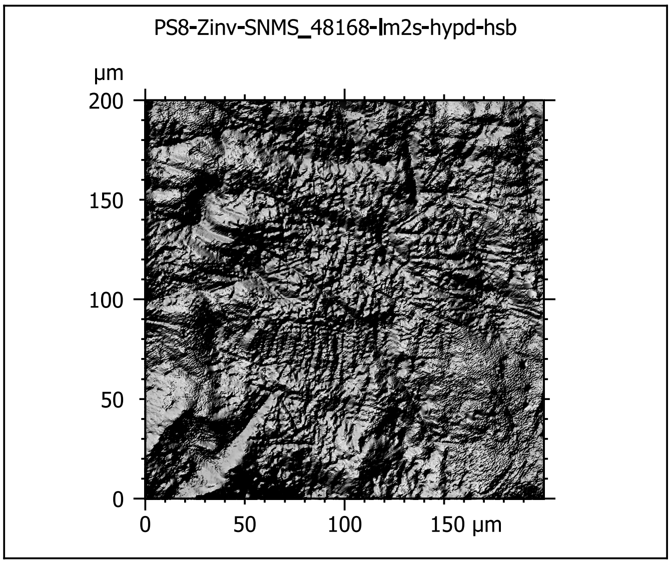

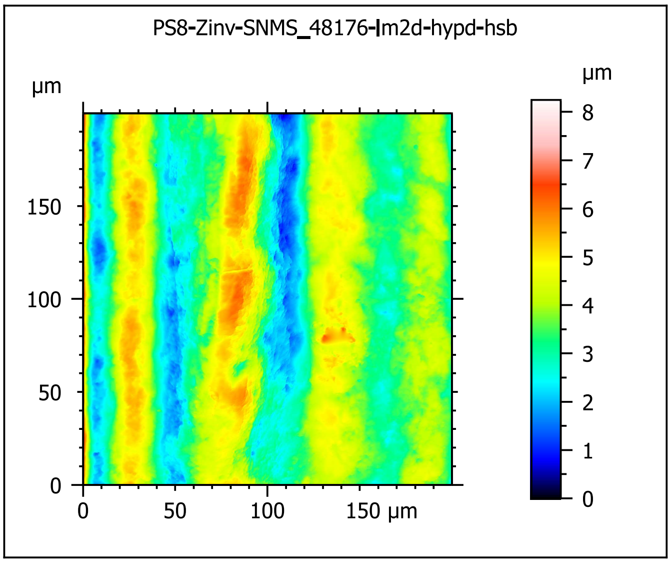

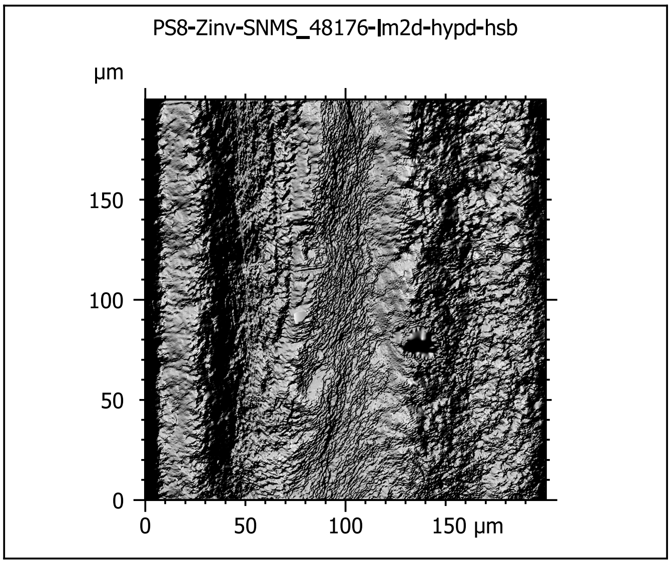

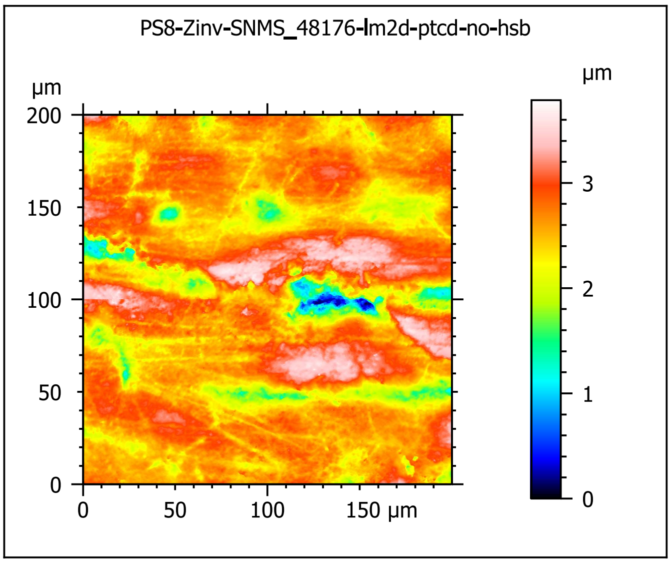

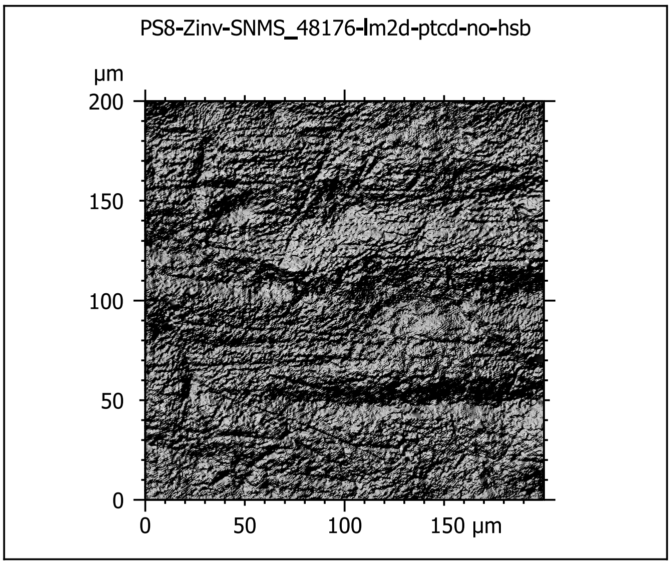

MountainsMap® Premium 10.0.10510
